# Perceptions of problem-drinker patients’ family members about their own hazardous-drinking behaviours in Chinese general hospitals: a qualitative study

**DOI:** 10.1186/s12888-017-1348-5

**Published:** 2017-05-18

**Authors:** Chen-Chun Lin, Yun-Fang Tsai, Wen-Ling Yeh, Jung-Ta Kao, Ching-Yen Chen

**Affiliations:** 1 Department of Hepato-Gastroenterology, Chang Gung Memorial Hospital at Linkou, Tao-Yuan, Taiwan; 2 School of Nursing, College of Medicine, Chang Gung University, Tao-Yuan, Taiwan; 3 Department of Nursing, Chang Gung University of Science and Technology, Tao-Yuan, Taiwan; 4 Department of Psychiatry, Chang Gung Memorial Hospital at Keelung, Keelung, Taiwan; 5 Department of Traumatology Orthopedics, Chang Gung Memorial Hospital at Linkou, Tao-Yuan, Taiwan; 6 School of Medicine, College of Medicine, China Medical University, Taichung, Taiwan; 7 Department of Internal Medicine, Division of Hepato-Gastroenterology, China Medical University Hospital, Taichung, Taiwan; 8 College of Medicine, Chang Gung University, Tao-Yuan, Taiwan

**Keywords:** Alcohol, Family members, Hazardous drinker, Drinking behaviours

## Abstract

**Background:**

Excessive alcohol use has been associated with health, social and legal problems. Alcohol-related problems have been studied primarily in problem-drinker patients, with few studies on their family members, particularly about their own hazardous or harmful alcohol-drinking behaviours.

**Method:**

In this qualitative descriptive study, participants were recruited from three hospitals randomly selected from northern and central Taiwan (2:1). Hazardous-drinker patients and their family members were screened using the Chinese version Alcohol Use Disorders Identification Test (scores ≥8 indicate harmful or hazardous drinkers). Data were collected in individual, audiotaped, in-depth interviews using an interview guide. Verbatim interview transcripts were analysed using ATLAS.ti, version WIN 7.0.

**Results:**

The sample of 35 family members with hazardous or harmful drinking behaviours perceived that their own alcohol-drinking behaviours were related to six major patterns: family habits, leisure activities with friends, work pressures, personal taste, a way to forget one’s problems and to express happiness.

**Conclusion:**

We recommend that programmes targeting harmful or hazardous drinking among problem-drinker patients’ family members should educate participants about the standard amounts of alcohol in alcoholic beverages, recommended amounts of alcohol consumption for males and females, the long-term effects of excessive alcohol consumption; address sources of risk factors at work; offer strategies to resist social pressures to drink; and build positive strategies for coping with stress.

## Background

Excessive alcohol use has been associated with a large variety of health, social, and legal problems [1]. Individuals with a family history of alcoholism are at greater risk of developing alcoholism than those without such a history [2]. Moreover, the link between parental problem drinking and children’s maladjustment is well established [3]. Thus, alcoholism and its effects on family members create a vicious cycle that should be interrupted. In addition, close family members of patients with alcohol problems may suffer from stress-related physical and psychological symptoms that can be severe and long lasting [4, 5] and are associated with high use of primary care services [6], placing a significant burden on healthcare resources [7]. These factors highlight the importance of screening and intervening for problem drinking in family members of patients identified as problem drinkers.

One appropriate setting suggested for detecting hazardous or harmful drinkers is general hospitals [8]. In general hospitals in western countries, the prevalence of patients’ alcohol-drinking problems ranges from 12% to 26% due to different assessment methods and units selected [8, 9]. However, no information is available for the prevalence of alcohol-drinking problems among family members of these patients in western countries. Moreover, current studies of alcohol-related healthcare problems in western countries have mainly focused on patients, with few studies on their family members [10, 11].

Alcohol, which is legally accessible in Taiwan, plays an important role in Chinese culture as it is viewed as an acceptable drink to relieve stress and enhance socialization [12]. As a result, drinking problems are easily ignored. In Taiwan’s general hospitals, where the prevalence of patients’ alcohol-drinking problems ranges from 5.7% to 19.2% due to different units selected [13, 14]. The prevalence of hazardous alcohol-drinking problems among family members of patients with alcohol-drinking problems in Taiwan was 13.3% [15]. As in western countries, alcohol-drinking problems in Taiwan have mainly been studied in problem-drinker patients, with few studies on their family members [15–18]. Among these studies, the focus was couple relationships and alcohol use [16, 17]. Only one study also explored spouses of alcoholic patients for their stressors, coping behaviours, and mental health status [18].

In the only study found on family members of Chinese problem-drinker patients, their risk factors for hazardous alcohol-drinking problems were explored, along with previous assessments and interventions for alcohol-drinking problems [15]. That study found that these family members’ risk factors for hazardous drinking were male gender, low education level, heart disease, smoking, and chewing betel quid. Only 11.8% of participants had been assessed for drinking problems in the previous year, and only 37.7% of participants with drinking problems had received a drinking intervention in the previous year. The authors concluded that alcohol problems among family members of problem-drinker patients in Taiwanese general hospitals are insufficiently assessed and targeted with interventions [15]. That study was limited by assessing only demographic data and illness-related variables, which are not changeable. Thus, the only modifiable risk factors identified were smoking and chewing betel quid.

Detecting and intervening with hazardous or harmful drinkers is important because these drinkers’ behaviours negatively affect family members, highlighting the need to design suitable interventions to prevent them from hazardous drinking. Modifiable risk factors must be targeted for interventions to effectively reduce hazardous alcohol-drinking behaviours among family members of problem-drinker patients. Important mediators of behaviour, identified from cognitive [19] and cognitive-behavioural [20] approaches are cognitive patterns, e.g. beliefs, expectations, styles, or capabilities. For example, one’s beliefs and hopeless expectancies are related to whether one attempts or completes suicide [19, 21]. Since the beliefs and expectations of hazardous alcohol drinkers about hazardous alcohol-drinking behaviours may be considered risk factors for hazardous alcohol-drinking behaviours, they are important to understand. Understanding these factors can help nurses and clinicians prevent hazardous alcohol-drinking behaviours in family members of problem-drinker patients by designing interventions to decrease risk factors for hazardous alcohol-drinking behaviours. Therefore, the purpose of this study was to explore the perceptions of family members of problem-drinker patients about their own hazardous or harmful alcohol-drinking behaviours.

## Method

### Design

This qualitative descriptive study was part of a large series of studies to establish a model of hazardous-drinking behaviours among family members of hazardous-drinker patients in Taiwan. This paper follows the Consolidated Criteria for Reporting Qualitative Research (COREQ) [22].

### Sample and setting

A representative sample of family members of hazardous-drinker patients was ensured by recruiting these patients from psychiatric, gastrointestinal medical-surgical, trauma, and rehabilitation clinics and wards where most patients with alcohol problems are seen in Taiwan [23]. Since our research team is working in northern and central Taiwan, we chose these two areas to conduct our study. Northern and central Taiwan have 127 and 105 accredited medical centres, regional, and district hospitals, respectively [24]. Therefore, three hospitals were randomly selected from northern and central Taiwan in a 2:1 ratio. All hazardous-drinker patients at the selected hospitals were referred by physicians or nurses. Patients were asked to refer their family members to participate in this study if patients met these criteria: AUDIT score ≥ 8, > 20 years old, and able to speak Chinese/Taiwanese. Family members were included in the original study if they met these criteria: 1) > 20 years old, 2) parent, sibling, child, or partner of a hazardous-drinker patient, and 3) able to speak Chinese/Taiwanese. Family members were divided into two groups (AUDIT score < 8 and ≥8). Only data of family members with AUDIT scores ≥8 were used in this study. Participants were recruited until analysis of interview data revealed no new findings (data saturation).

### Data collection

Quantitative data on hazardous-drinker patients’ and their family members’ alcohol drinking-related behaviours were collected using the Chinese-version Alcohol Use Disorders Identification Test (AUDIT) [23]. The 10-item AUDIT measures alcohol consumption level (3 items), symptoms of alcohol dependence (3 items), and problems associated with alcohol use (4 items) in the previous year. Higher AUDIT scores indicate more severe level of risk; scores ≥8 indicate a tendency to hazardous drinking.

Quantitative data on hazardous-drinker patients’ demographic information were collected using a researcher-designed form. A similar form was used to collect the same data from these patients’ family members plus two added items: ‘Relationship to the hazardous-drinker patient’ and ‘Are you living with the patient?’

Qualitative data were collected in individual in-depth interviews using an interview guide. Sample questions for all family members of hazardous-drinker patients were ‘What is your lived experience with your relative who has drinking problems? Can you describe the most memorable event regarding your relative’s alcohol drinking? How did his/her drinking behaviours interfere with your life?’ Family members with AUDIT scores ≥8 were further asked ‘Why have you chosen to use alcohol? Have you tried to use other substances?’ Interviews were audiotaped with participants’ permission. This paper focuses on participants’ responses to the question, ‘Why have you chosen to use alcohol?’

The content validities of the interview guide and researcher-designed demographic information form were explored by a panel of 10 experts in psychiatric care: five physicians with expertise in treating hazardous-drinker patients and five clinical nurses with extensive experience caring for hazardous-drinker patients. All members of the panel agreed that both forms were suitable to use in this population.

### Procedure

This study was carried out in accordance with the Code of Ethics of the World Medical Association. Each selected hospital was approached individually. After the hospitals’ institutional review boards approved the study, physicians or nurses in psychiatric, gastrointestinal medical-surgical, trauma, and rehabilitation clinics and wards were asked to refer hazardous-drinker patients. A research assistant (RA) approached referred cases, described the study, screened them for the inclusion criteria, obtained their written consent to participate, and asked them to refer their family members to participate in this study. These patients were also asked to fill out the researcher-designed form for demographics. The RA then approached family members, described the study, screened them for inclusion criteria, obtained written consent to participate, and collected data.

### Data analysis

All audiotapes were transcribed verbatim as soon as possible after interviews. Transcripts were analysed following Van Manen’s [25] steps of thematic analysis, i.e. turning to the nature of the experience, formulating the phenomenological questions, explicating assumptions and preunderstandings, exploring the phenomenon-generating data, consulting the phenomenological literature, conducting thematic analysis, determining essential themes, attending to how language is spoken, varying examples and writing. To help make sense of the large amount of qualitative information, we also used ATLAS.ti, version WIN 7.0, a programme for text analysis and model building (Atlas.ti Scientific Software Development GmbH, Germany). Participants’ demographic information was analysed by descriptive statistics (frequencies, percentages, means, and standard deviations).

## Results

### Participants’ characteristics

Of 120 patients approached, 114 agreed to participate. The remaining 6 patients refused due to time constraints. Of the 114 family members referred by patient participants, 4 refused to participate due to time constraints, and 5 could not be reached. Among these 105 family members recruited for the study, 35 had AUDIT scores ≥8. These 35 family members had a mean age of 44.2 (SD = 11.9, range = 28–68) and a mean AUDIT score of 11.2 (SD = 2.7, range = 8–18). The majority was male (57.1%), married (74.3%), lived with a partner and children (51.4%), had not smoked in the past year (60%), had not chewed betel quid (85.7%) in the past year, and lived with the patients (68.6%). Their occupations varied. The majority of family-member participants drank more than one type of alcoholic beverage (51.4%), with the most popular beverage being beer (82.9%). The greatest proportion was the husband/wife of the hazardous-drinker patient (45.7%) (Table 1).

**Table 1:** Demographic characteristics of problem drinkers’ family members ( *N* = 35)

Characteristic	*n* (%)	mean (SD)
Age (years)		44.2 (11.9)
Gender		
Male	20 (57.1)	
Female	15 (42.9)	
Marital status		
Single	7 (20.0)	
Married	26 (74.3)	
Divorced	2 (5.7)	
Occupation		
Farmer	2 (5.7)	
Plasterer/plumber/electrician	7 (20.0)	
Administrative clerk/manager	9 (25.7)	
Driver	2 (5.7)	
Freelancer (e.g., writer)	2 (5.7)	
Service industry	12 (34.3)	
Housewife	3 (8.6)	
Retired	2 (5.7)	
Living status		
With partner	6 (17.1)	
With child	2 (5.7)	
With partner and child	18 (51.4)	
With parents	6 (17.1)	
With parents, partner and child	1 (2.9)	
With parents, sibling, partner and child	1 (2.9)	
With mother	1 (2.9)	
Number of children		1.7 (1.3)
Smoked in the past year		
No	21 (60.0)	
Yes	114 (40.0)	
Chewed betel quid in the past year		
No	30 (85.7)	
Yes	5 (14.3)	
Common alcoholic beverages		
Beer	14 (40.0)	
Sorghum wine	1 (2.9)	
Rice wine	1 (2.9)	
Whiskey	1 (2.9)	
Beer + sorghum wine	4 (11.4)	
Beer + whiskey	5 (14.3)	
Beer + red wine	3 (8.6)	
Sorghum wine + rice wine	2 (5.7)	
Red wine + whiskey	1 (2.9)	
Beer + whiskey + red wine	1 (2.9)	
Beer + sorghum wine + red wine	1 (2.9)	
Beer + whiskey + sorghum wine + brandy	1 (2.9)	
Drinking behaviours in the past month		
Number of drinks/week		2.5 (1.9)
Number of binges		1.5 (1.7)
Number of standard drinks/binge, mean (SD)		6.3 (3.7)
Relationship to the hazardous-drinker patient		
Parent	1 (2.9)	
Sibling	7 (20.0)	
Child	11 (31.4)	
Husband/wife	16 (45.7)	
Living with the patient		
No	11 (31.4)	
Yes	24 (68.6)	

The mean age of the 35 hazardous-drinker patients related to family-member participants was 51.3 (SD = 14.2, range = 28–82), and their mean AUDIT score was 12.4 (SD = 4.4, range = 8–26). The majority was male (60%), married (71.4%), had smoked in the past year (51.4%), but had not chewed betel quid (71.4%) in the past year (Table 2).

**Table 2:** Demographic characteristics of problem-drinker patients ( *N* = 35)

Characteristic	n (%)	mean (SD)
Age (years)		51.3 (14.2)
Gender		
Male	21 (60.0)	
Female	14 (40.0)	
Marital status		
Single	6 (17.1)	
Married	25 (71.4)	
Divorced	2 (5.7)	
Widow/Widower	2 (5.7)	
Living status		
Alone	3 (8.6)	
With partner	10 (28.6)	
With child	3 (8.6)	
With partner and child	16 (45.7)	
With parents	3 (8.6)	
Number of children		2.1 (1.5)
Smoked in the past year		
No	17 (48.6)	
Yes	18 (51.4)	
Chewed betel quid in the past year		
No	25 (71.4)	
Yes	10 (28.6)	
Drinking behaviours in the past month		
Number of drinks/week		2.9 (2.2)
Number of binges		1.6 (2.0)
Number of standard drinks/binge		6.7 (5.8)

### Family members’ perceptions of their own hazardous or harmful alcohol-drinking behaviours

Family members’ perceptions of their own alcohol-drinking behaviours were related to six major patterns: family habits, leisure activities with friends, work pressures, personal taste, a way to forget one’s problems and to express happiness. These findings are presented in detail below with representative quotes from interview transcripts.

#### Family habits

Several participants mentioned that their family members were used to drinking together. They commonly drank during the dinner time or after dinner when they watched television. As F20 said, ‘Our family used to drink some wine during dinner. It’s our habit.’ Similarly, F75 said, ‘I used to drink when I watched TV with my husband. It’s very relaxing. I have got used to it. If we watch TV and don’t drink, we feel that something’s wrong.’

Many participants expressed how enjoyable this family habit was. As F39 said, ‘The feeling of drinking with my husband and children is good. We chat with each other while drinking. I enjoy the feeling of being together and very relaxed.’

Moreover, all participants emphasized that they did not drink too much. The amount of drinking was under their control. As F103 said, ‘We don’t over-drink. When we feel that we have drunk enough, we stop. We always stop before feeling drunk, so we can control the amount.’

Participants brought this family habit to their derivative family. As F93 said, ‘When I was at home, I used to drink with my father. I really enjoyed it. After I got married, I also drank after work. When my husband saw me drinking, he tried it. Now, we drink together.’

#### Leisure activities with friends

Many participants expressed that drinking was part of their leisure activities and time spent with their friends. Eating, drinking, singing and chatting with friends were ways for them to relax and enjoy themselves. As F80 said, ‘I work very long hours. My only leisure is to go out with my former classmates. We find a restaurant, have nice food and wine. It’s easy to get high. We talk and sing. Everyone feels happy.’ Similarly, F50 said,

My work has deadlines and is very stressful. After finishing a project, I call my friends and go to a night club or bar together. You know, we drink and make friends there. It’s a good way to unwind and have fun.

In addition, some participants were blue-collar workers who commonly drank with their co-workers after work. As F64 said, ‘I am a plasterer. My work team is used to drinking together after work. It’s a way to relax, particularly after a hot summer day. Drinking a cold beer is extremely good.’

#### Work pressures

Some participants expressed their dislike for drinking alcohol, but felt pressured to drink with their work clients. As F57 said, ‘I don’t like to drink, but in my work I need to entertain my clients. I need to drink 3–4 times per week. I don’t have any choice.’ Similarly, F100 said:

We own a stir-fry shop which sells food and drinks. The atmosphere is very fast paced and high spirited. Some of the customers are very enthusiastic and buy me a drink. I can’t say no. Therefore, I often drink several glasses every night.

#### Personal taste

Participants also expressed that they enjoyed tasting wine. They viewed drinking and tasting wine as gaining new knowledge about wine. As F84 said, ‘I enjoy tasting wine. I drink it slowly and enjoy the taste. It’s my habit and preference. I don’t drink too much, so I don’t get drunk.’ Similarly, F71 said, ‘Red wine is a good wine. I enjoy tasting wine. I can gain lots of new knowledge about red wine. Tasting wine also helps me to relax.’

#### A way to forget one’s problems

Some participants said that drinking alcohol helped them to forget all bad things. As F87 said, ‘I used to have fights with my wife every day. We handle things very differently. When we fight, I feel sad and start to drink. Drinking helps me to forget all my problems and disappointments.’ Similarly, F104 said, ‘My mother used to drink alcohol to relieve her stress and anguish. I thought I could try. Now, I also use this way to escape my worries.’

#### To express happiness

Some participants disagreed about drinking as a way to relieve their stress. They said that they drank because they felt happy. As F29 said, ‘We [aboriginals] drink because we feel happy. We don’t drink to relieve stress.’ Similarly, F51 said, ‘I drink because I feel happy. I won’t drink in a sad mood.’

## Discussion

This study is the first to explore the perceptions of family members of problem-drinker patients about their own hazardous or harmful alcohol-drinking behaviours. Our results revealed that these family members’ perceptions of their own alcohol-drinking behaviours were related to six major patterns: family habits, leisure activities with friends, work pressures, personal taste, a way to forget one’s problems and to express happiness. We would like to emphasize that our patients already had physical problems for which they came to hospital for treatment. Furthermore, none of these patients were referred by their family members for problems related to drinking behaviours nor did families consider it necessary to decrease the patients’ drinking. This lack of awareness about the dangers of patients’ hazardous drinking is consistent with findings that people in the UK lacked knowledge about the long-term harmful effects of alcohol drinking [26]. These findings suggest the need to highlight knowledge of the long-term negative effects of alcohol drinking in programmes to prevent harmful or hazardous drinking.

Family habits as a pattern of drinking alcohol were described as ways to promote family cohesion and communication as well as to relax. This finding is unique; we could not find any published report of family habits as a type of drinking behaviour. Moreover, the participants who described this pattern did not report the amount of alcohol consumed or number of drinks, but monitored their drinking by stopping before they felt drunk. Using drunkenness as a guideline, all of them perceived that their drinking behaviours were under their control.

Since drinking alcohol is a learned behaviour [26], children can learn from their parents, husbands can learn from their wives, and vice versa. These findings highlight the importance of family environment in influencing their members’ behaviours. Changing a drinking pattern as a family habit may not be easy because the pattern is associated with many positive feelings and interactions. However, it is important to inform family members about standard amounts of alcohol in drinks (one standard drink contains about 14 g of ethanol [27] and the recommended amounts for males and females. For men, low-risk drinking is defined as no more than 4 (standard) drinks on any single day and no more than 14 drinks per week; for women, low-risk drinking is no more than 3 drinks on any single day and no more than 7 drinks per week [27]. Without understanding hazardous or harmful alcohol drinking, it is impossible for people to grasp the risks associated with their drinking behaviours.

Our participants also described drinking alcohol as connected to leisure activities with friends, as consistently reported in the literature. For example, peer pressure was described by Dutch adolescents (12–17 years old) as playing a role in their alcohol drinking [28]. However, drinking alcohol was described by mid-life adults in the US (24–54 years old) [29] and UK (35–50 years old) [30] as a more controlled social behaviour but still an issue at times. Our participants who described this pattern of drinking could be taught to have fun with friends by pursuing other leisure activities besides drinking with friends, such as walking or hiking, cycling, or camping. Another approach to decreasing their drinking behaviours would be to teach them more positive coping strategies.

Another pattern in our participants’ drinking behaviours was work pressures, consistent with 1172 US adults’ drinking behaviours being correlated with social pressures to drink [31]. Our participants with this pattern of drinking could be taught strategies to resist social and work pressures, which may help to change their drinking behaviours.

Personal taste was another drinking pattern related to tasting wine, which has recently become fashionable in Taiwan. People feel that it is a gracious habit that expresses a sophisticated and cosmopolitan lifestyle. Red wine in small amounts has also been reported as good for health [32]. Unfortunately, these participants did not report the amount of wine they drank, but monitored their alcohol consumption by not feeling drunk, as reported by participants who described the family habits pattern. Therefore, prevention programme s for these participants should also consider including the standard amounts of alcohol in different kinds of drinks, e.g. one standard drink equals 12 oz of beer, 5 oz of wine, or 1.5 oz of spirits [27], the recommended alcohol amounts for males and females, and knowledge about the long-term effects of hazardous or harmful alcohol drinking.

Drinking alcohol was also considered by our participants as helping them to forget their problems, which may demonstrate a maladaptive pattern. Like the family-habit pattern, this pattern was described by our participants as a behaviour learned from the family environment. These findings highlight the importance of family education about drinking alcohol and helping people with this drinking pattern to build positive coping strategies to deal with their problems. In contrast, one group of our participants described drinking alcohol when they felt happy. Drinking for these participants was related to celebration and happiness. Programmes for these participants should focus on educating them about the standard measures of alcoholic beverages, the recommended alcohol amounts for males and females, and the long-term effects of hazardous or harmful alcohol drinking.

### Limitations

Although this study provides important information about the perceptions of family members of problem-drinker patients about their own hazardous or harmful alcohol-drinking behaviours, it had two limitations. The sample was recruited by convenience from three randomly selected hospitals in northern and central Taiwan. Thus, participants’ opinions may not represent the perceptions of family members of problem-drinker patients from other parts of Taiwan or a randomly chosen sample. Future studies should use random sampling and expand data collection settings.

## Conclusion

Excessive alcohol use has been associated with a large variety of health, social, and legal problems [1]. This study found that family members of problem-drinker patients perceived that their own alcohol-drinking behaviours were related to six major patterns: family habits, leisure activities with friends, work pressures, personal taste, a way to forget one’s problems, and to express happiness. Our findings highlight the importance of developing intervention programmes for this understudied population. Future intervention programmes for family members of hazardous or harmful drinkers should emphasize educating about the standard measures of alcoholic beverages, the recommended alcohol amounts for males and females, the long-term effects of hazardous or harmful alcohol drinking; address sources of risk factors at work; offer strategies to resist social pressure to drink; and build positive strategies to cope with stress. Moreover, our experience may serve as a reference for developing alcohol-drinking prevention programmes for hazardous drinking behaviours among family members of problem-drinker patients.

## References

[1] World Health Organization. Management of Substance Abuse. Facts and Figures: Alcohol, 2009. http://www.who.int/substance_abuse/facts/alcohol/en/index.html (5 July 2016, date last accessed).

[2] Rojas JI, Nisbet RB (2011). Detecting alcohol use disorders in primary care settings. J Okla State Med Assoc.

[3] Keller PS, Cummings EM, Davies PT, Mitchell PM (2008). Longitudinal relations between parental drinking problems, family functioning, and child adjustment. Dev Psychopathol.

[4] Copello A, Templeton L, Krishnan M, Orford J, Velleman R (2000). A treatment package to improve primary care services for relatives of people with alcohol and drug problems. Addict Re Theory.

[5] Orford J, Natera G, Davies J, Nava A, Mora J, Rigby K (1998). Tolerate, engage or withdraw: a study of the structure of families coping with alcohol and drug problems in south west England and Mexico City. Addict.

[6] Svenson LW, Forster DI, Woodhead SE, Platt GH (1995). Individuals with a chemical-dependent family member. Does their health care use increase?. Can Fam Physician.

[7] Ray GT, Mertens JR, Weisner C (2007). The excess medical cost and health problems of family members of persons diagnosed with alcohol or drug problems. Med Care.

[8] Roche AM, Freeman T, Skinner N (2006). From data to evidence, to action: findings from a systematic review of hospital screening studies for high risk alcohol consumption. Drug Alcohol Depend.

[9] Rosón B, Monte R, Gamallo R, Puerta R, Zapatero A, Fernández-Solá J (2010). Prevalence and routine assessment of unhealthy alcohol use in hospitalized patients. Eur J Intern Med.

[10] Copello A, Templeton L, Orford J, Velleman R, Patel A, Moore L (2009). The relative efficacy of two levels of a primary care intervention for family members affected by the addiction problem of a close relative: a randomized trial. Addict.

[11] Orford J, Velleman R, Natera G, Templeton L, Copello A (2013). Addiction in the family is a major but neglected contributor to the global burden of adult ill-health. Soc Sci Med.

[12] Li YC, Wang FL (1996). Alcohol abuse and the family of alcoholism. Med Digest.

[13] Tsai YF, Tsai MC, Lin YP, Chen CY (2009). Brief intervention for problem drinkers in a Chinese population: a randomized controlled trial in a hospital setting. Alcohol Clin Exp Res.

[14] Tsai YF, Lin YP, Tsai MC, Weng CE, Chen CY (2013). Hazardous alcohol-drinking problems among a Chinese hospital patient population. J Adv Nurs.

[15] Tsai YF, Chen CY, Lin YP, Tsai MC, Weng CE (2012). Hazardous drinking problems in family members of problem-drinker patients in Chinese general hospitals. Gen Hosp Psychiatry.

[16] Chung CW. The Research of Alcoholics' Marriage: From Couples' Viewpoints. Unpublished master’s thesis, National Changhua University of Education: Changhua, Taiwan, 2005.

[17] Kuo YC. Marital Struggle among Wives of Alcoholics. Unpublished master’s thesis, Tunghai University: Taichung, Taiwan, 2008.

[18] Su IC. An Exploratory Study of Stressors, Coping Behaviors and Mental Health Status among Spouses of Alcoholic Patients. Unpublished master’s thesis, Kaohsiung Medical College: Kaohsiung, Taiwan, 1994.

[19] Beck AT (1963). Thinking and depression. I. Idiosyncratic content and cognitive distortions. Arch Gen Psychiatry.

[20] Linehan MM, Nielsen SL (1981). Assessment of suicide ideation and parasuicide: hopelessness and social desirability. J Consult Clin Psychol.

[21] Bedrosian RC, Beck AT (1979). Cognitive aspects of suicidal behavior. Suicide Life Threat Behav.

[22] Tong A, Sainsbury P, Craig J (2007). Consolidated criteria for reporting qualitative research (COREQ): a 32-item checklist for interviews and focus groups. Int J Qual Health Care.

[23] Tsai MC, Tsai YF, Chen CY, Liu CY (2005). Alcohol use disorders identification test (AUDIT): establishment of cut-off scores in a hospitalized Chinese population. Alcohol Clin Exp Res.

[24] Ministry of Health and Welfare. 2013 to 2016 Years of Hospital Evaluation and Teaching Hospital Evaluation of Qualified List. http://dep.mohw.gov.tw/DOMA/cp-949-15569-106.html. Accessed 12 Apr 2017.

[25] Van Manen M. Researching Lived Experience: Human Science for an Action Sensitive Pedagogy. State University of New York Press, Albany, 1990.

[26] Jacob N, MacArthur GJ, Hickman M, Campbell R (2016). A qualitative investigation of the role of the family in structuring young people's alcohol use. Eur J Pub Health.

[27] The U.S. National Institute on Alcohol Abuse and Alcoholism. Alcohol & Your Health, 2016*.* https://www.niaaa.nih.gov/alcohol-health (5 July 2016, date accessed).

[28] Janssen MM, Mathijssen JJ, van Bon-Martens MJ, van Oers HA, Garretsen HF (2014). A qualitative exploration of attitudes towards alcohol, and the role of parents and peers of two alcohol-attitude-based segments of the adolescent population. Subst Abuse Treat Prev Policy.

[29] Wolf JP, Chávez R (2015). "just make sure you can get up and parent the next day": understanding the contexts, risks, and rewards of alcohol consumption for parents. Fam Soc.

[30] Emslie C, Hunt K, Lyons A (2012). Older and wiser? Men's and women's accounts of drinking in early mid-life. Sociol Health Illn.

[31] Dawson DA, Goldstein RB, Ruan WJ, Grant BF (2012). Correlates of recovery from alcohol dependence: a prospective study over a 3-year follow-up interval. Alcohol Clin Exp Res.

[32] Lagrue-Lak-Hal AH, Andriantsitohaina R (2006). Red wine and cardiovascular risks. Arch Mal Coeur Vaiss.

